# Neglected Tropical Diseases: A Systematic Evaluation of Research Capacity in Nigeria

**DOI:** 10.1371/journal.pntd.0003078

**Published:** 2014-08-14

**Authors:** Patricia N. Okorie, Moses J. Bockarie, David H. Molyneux, Louise A. Kelly-Hope

**Affiliations:** 1 Institute for Advanced Medical Research and Training, College of Medicine, University of Ibadan, Ibadan, Nigeria; 2 Centre for Neglected Tropical Diseases, Department of Parasitology, Liverpool School of Tropical Medicine, Liverpool, United Kingdom; University of Agriculture, Nigeria

## Abstract

**Background:**

Nigeria carries the highest burden and diversity of neglected tropical diseases (NTDs) in sub-Saharan Africa and is preparing to scale up its efforts to control/eliminate these diseases. To achieve this it will require a range of internal technical support and expertise for mapping, monitoring and evaluating, operational research and documenting its success. In order to begin to evaluate this potential in Nigeria, this study collated and analysed information for lymphatic filariasis (LF), onchocerciasis, schistosomiasis and soil-transmitted helminths (STH), which are currently being targeted with preventive chemotherapy through mass drug administration (MDA).

**Methodology/Principal Findings:**

Information from 299 scientific articles published on the selected NTDs in 179 journals between January 2008 and September 2013 was extracted and systematically compiled into a geo-referenced database for analysis and mapping. The highest number of articles was from the southern geo-political zones of the country. The majority of articles focused on one specific disease, and schistosomiasis and STH were found to have the highest and most wide ranging research output. The main type of study was parasitological, and the least was biotechnological. Nigerian authors were mostly affiliated with universities, and there was a wide range of international co-authors from Africa and other regions, especially the USA and UK. The majority of articles were published in journals with no known impact factor.

**Conclusions/Significance:**

The extensive database and series of maps on the research capacity within Nigeria produced in this study highlights the current potential that exists, and needs to be fully maximized for the control/elimination of NTDs in the country. This study provides an important model approach that can be applied to other low and middle income countries where NTDs are endemic, and NTD programmes require support from the expertise within their own country, as well as internationally, to help raise their profile and importance.

## Author Summary

Nigeria carries the highest burden and diversity of neglected tropical diseases (NTDs) in sub-Saharan Africa and is preparing to increase the control and elimination of these diseases. The aim of this study was to provide information on the disease focus and type of studies carried out by scientists working on lymphatic filariasis (LF), onchocerciasis, schistosomiasis, soil-transmitted helminths (STH)) and *Loa loa* filariasis in Nigeria. Information on these diseases from all published literature on the scientific articles by Nigerian authors published between January 2008 and September 2013 was collated and mapped. The results show that many institutions are working on NTDs in Nigeria and their capacity could be readily enhanced with training and resources to boost their skills and to increase their range of technical activities and research visibility, which will also help to provide essential technical and laboratory support to the national NTD programmes.

## Introduction

The impact of neglected tropical diseases (NTDs) on the health and economy of neglected communities is gaining increasing international attention with a call for global efforts to eliminate or eradicate 10 NTDs by 2020 [1] [2]. NTDs are a group of infections that mainly affect people living in remote rural areas, urban slums or conflict zones [2]. In recent years, international funding for NTD control and elimination has increased through support by several donors including pharmaceutical companies, governments, UK Department for International Development (DFID), the United States Agency for International Development (USAID), Bill & Melinda Gates Foundation, World Bank, World Health Organization (WHO), non-governmental development organizations (NGDOs) and other research agencies [1] [3]–[5].There is a need to build on this momentum but a key issue, which has been highlighted, including in the WHA Resolution, is the need for increased research efforts and for strengthening capacity in endemic countries for both research and implementation.

However, financial support has not been evenly distributed with some countries overwhelmed with significant donor support, while other countries with an equally large burden of diseases have attracted little or no funding [6]. This increase in funding may be more appropriately and evenly channelled if potential synergies between different disease programmes were better identified. To date NTD programmes have not fully exploited the benefits of these synergies [7] [8], despite many of the diseases being co-endemic. The delivery systems could be shared thus making the interventions for NTDs more cost effective [7] [9]. NTD programmes can be integrated into primary health care services and existing vaccination or micronutrient campaigns, or the school based distribution of anthelminthic drugs to achieve greater coverage and reduce operational costs [4]. The opportunities to maximise the benefits of one programme on another abounds. For example, the potential benefit of repeated doses of ivermectin for onchocerciasis on the prevalence of *Wuchereria bancrofti* (LF) [10] [11], and the combination of ivermectin and albendazole for LF on soil-transmitted helminths (STH) prevalence and scabies [1] [12] have been demonstrated.

In Nigeria, the opportunities and importance of scaling up interventions in a coordinated way across NTD programmes has recently been highlighted by Okorie and others [9] and in the five-year NTD Master Plan launched by the Federal Ministry of Health (FMoH) [13]. The NTD programmes are currently delivered through the State Ministries of Health, and the Local Government Authorities following the technical recommendations from the FMoH. The need for strong technical knowledge as well as managerial skills at the State and local level is essential in order to train and supervise the various cadres of workers, organize appropriate health education, and monitor and evaluate the outcomes. This can most effectively be achieved through strong partnership and collaborative links with the different sectors within the country, including academia, which may be able to provide a range of in-country technical support and expertise for mapping, monitoring and evaluating, operational research and documenting programmatic success.

The availability of technical capacity, in terms of research scientists and institutions, in developing countries is a major challenge for the control of NTDs [1] [14]. The need for developing and supporting this capacity within NTD endemic countries cannot be over emphasized as it would guarantee national ownership and help to make the programmes accountable and sustainable [2] [5]. Although the FMoH in Nigeria have a lot of field capacity at state and local government level that could be utilized, additional support is required from the researchers within the country to execute its projects. In order to begin to evaluate this potential in Nigeria, and to develop a model approach for other countries, information from the published literature on the scientific research capacities for the select group of NTDs being targeted with preventive chemotherapy through mass drug administration (MDA) were collated and analysed [1].

This paper addresses the issue of country capacity for research as a resource to inform NTD programmes with reference to Nigeria, which carries the highest burden and diversity of NTDs in sub-Saharan Africa [15]. The NTDs most prevalent in Nigeria include lymphatic filariasis (LF), onchocerciasis, schistosomiaisis, STH, trachoma, leprosy, Buruli ulcer and human African trypanosomiasis (HAT) [13]. This study however, focused on the diseases amenable to MDA including LF, onchocerciasis, schistosomiasis, STH as well as *Loa loa* filariasis. The latter is not officially classified as an NTD, but poses a major problem for LF and onchocerciasis programmes in co-endemic areas due to the risk of severe adverse events associated with the use of ivermectin [16].

## Methods

### Search strategy

A systematic search for published articles on the selected NTDs was conducted using electronic sources including PubMed, BiomedExperts, Google Scholar, Google and African Journal Online (AJOL). Search terms included Nigeria, in combination with each of the diseases, including alternative names of the disease, and the parasites; lymphatic filariasis, (elephantiasis), *Wuchereria bancrofti*, onchocerciasis (river blindness), *Onchocerca volvulus*, schistosomiasis, *Loa loa* (loiasis) and soil-transmitted helminths (Ascariasis, Taeniasis, Trichuriasis and Hookworm). Additional references were identified within the collated articles, and then from the references within those articles. Websites of the various institutions in Nigeria (when available) were also searched for the publication list of scientists in related departments. All articles with Nigerian authors and those published between January 2008 and September 2013 were included in the study to provide information and perspective on the most recent research capacity and activity. This information on researchers who are currently active will be useful to many internal and international partners, donors, and scientists who wish to collaborate with NTD researchers in country.

### Details of articles

The following information was extracted from each article into a data base i) article authors and title ii) publication year iii) disease focus and iv) journal name. Additional information on the journal and author composition was entered into the database and included v) journal website if available, vi) journal impact factor based on the Journal Citation Reports (JCR) of 2013, journal website or Researchgate website (www.researchgate.net) and vii) number of Nigerian authors, viii) number of international authors and ix) the country of international co/authors. Based on the lead authors' institution, information on the location was also entered for mapping purposes and included x) geopolitical zone xi) state xii) place (town, city), and xiii) geographical coordinates (latitude, longitude) of the location of the institution.

### Classification of studies and institutions

To understand the range of studies being conducted across the country, the type of study of each article was broadly classified based on the material and methods described, and included the following; parasitological (if parasitological techniques were used); entomological (if the study had a vector component); social/anthropological (if the study included the use of questionnaire, focus group discussion and/or observations); clinical/physical examination (if the participants of the study were subjected to clinical examination for the pathology of the disease); hospital based (if the study was conducted in a hospital); biotechnological (if molecular techniques were used) and review (if the disease or epidemiological aspects were reviewed). Multiple categories could be included in the database.

Further, to understand the range of institutions involved in NTD research in Nigeria, the type of institution was broadly classified based on the lead author's affiliation, and included the following; University, Research Institute, College/Polytechnic (including colleges of health and education, polytechnics, schools of technology), Diagnostic laboratory, Hospital, Ministry of Health, and Non-government/Development Organizations (NGO/NGDOs).

### Data analysis

Data were entered into and examined using univariate and bivariate tabulations in Excel (Microsoft office 2007), and mapped using the geographical information systems software ArcGIS 10 (ESRI, Redlands, CA). First, the number of articles were quantified by year and disease focus, and mapped by state and geo-political zone and to highlight areas where multiple diseases were being researched. Second, the type of study and institutional affiliation associated with each article was examined by disease focus, and then mapped to highlight the distribution of the different technical and academic capabilities and institutional foci available across the country. Third, the number of international co-authors, by disease focus and their country of origin were examined to identify international collaborative trends. Finally, the number of different journals and their impact factors were examined to determine if the disease focus and international collaboration had an influence on publication trends and research exposure.

## Results

### Number of NTD publications by year, disease focus and location

**Figure 1:**
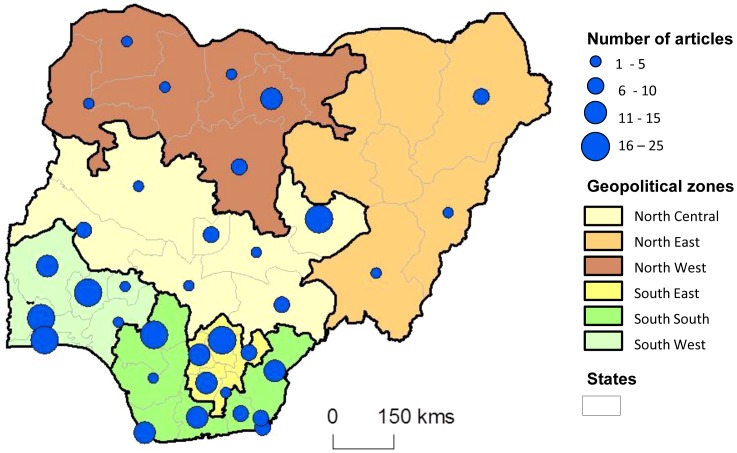
Map of total number of articles published by state and geopolitical zone.

A total of 299 published articles on LF, onchocerciasis, schistosomiasis, STH and *L. loa* between January 2008 and September 2013 were identified and collated into a database (Table S1). The number of articles published per year ranged from 35 to 58, with an average of 53 articles per year (Table 1). Based on the lead author's institution location, all six zones of the country had published on the NTDs and there were articles from 31 States and the Federal Capital Territory (Table S1). No articles on these NTDs were published from Bayelsa, Bauchi, Gombe, Yobe and Jigawa States. The highest number of articles were from the southern geo-political zones in the South West (n = 71), South East (n = 63) and South South (n = 60), and from states within these zones including Edo State (n = 25), Enugu State (n = 22) and Ogun State (n = 19) as shown in Figure 1.

**Table 1:** Number of publications by year for each main disease focus.

Disease focus	2008	2009	2010	2011	2012	2013*	Total
LF	5	3	7	14	7	5	**41**
Onchocerciasis	15	6	14	10	9	4	**58**
Schistosomiasis	15	23	17	13	24	13	**105**
STH	12	11	19	19	10	10	**81**
Loa loa	3	1	1	1	0	0	**6**
LF, Loa loa, Onchocerciasis	0	0	0	0	0	1	**1**
Schistosomiasis, STH	0	1	0	1	3	2	**7**
**Total**	**50**	**45**	**58**	**58**	**53**	**35**	**299**

* Total articles until September 2013 only (9 months).

**Figure 2:**
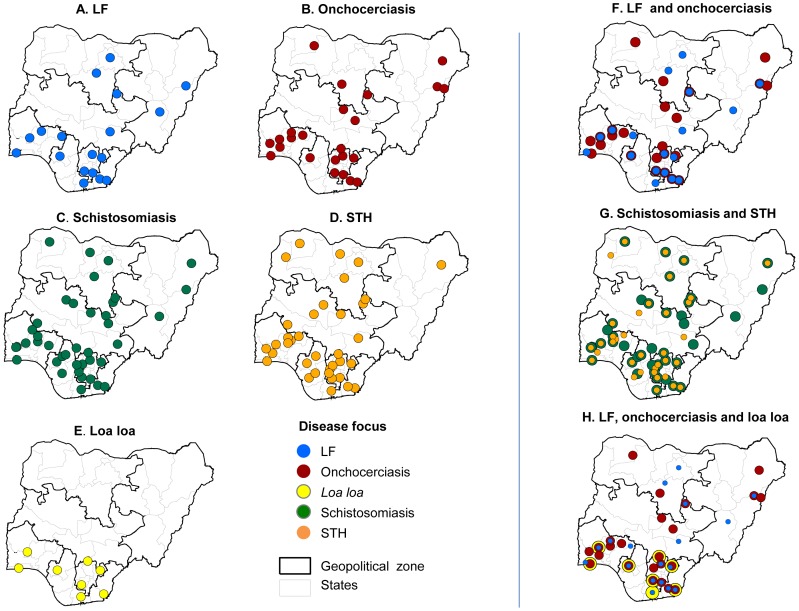
Institutional location of disease specific research. A. LF. B. Onchocerciasis. C. Schistosomiasis. D. STH. E. Loa loa. F. LF and onchocerciasis. G. Schistosomiasis and STH. H. LF, Onchocerciasis and Loa loa.

The number of publications varied by disease, with 41 articles on LF (13.7% of the total), 58 articles on onchocerciasis (19.4%), 107 articles on schistosomiasis (35.1%), 81 articles on STH (27.1%), and 6 articles on *L. loa* (2.0%). Only a small number of articles (n = 8; 2.7%) published on more than one disease (Table 1). There was no observable trend in the number of articles per year for any disease, however, most articles on LF were published in 2011 (n = 14), on onchocerciasis in 2010 (n = 14), on schistosomiasis in 2012 (n = 24), on STH in 2010 and 2011, on *L. loa* in 2008 (n = 3), and on multiple diseases between 2011 and 2013 (n = 7). The institutional location of where the disease specific research was carried out is shown in a series of maps in Figure 2a–e. Further overlapping maps were produced to highlight the locations and states where more than one NTD was being researched (Figure 2f–h), despite only a small number of articles being published on more than one disease.

### Type of study and institution by disease focus and location

**Figure 3:**
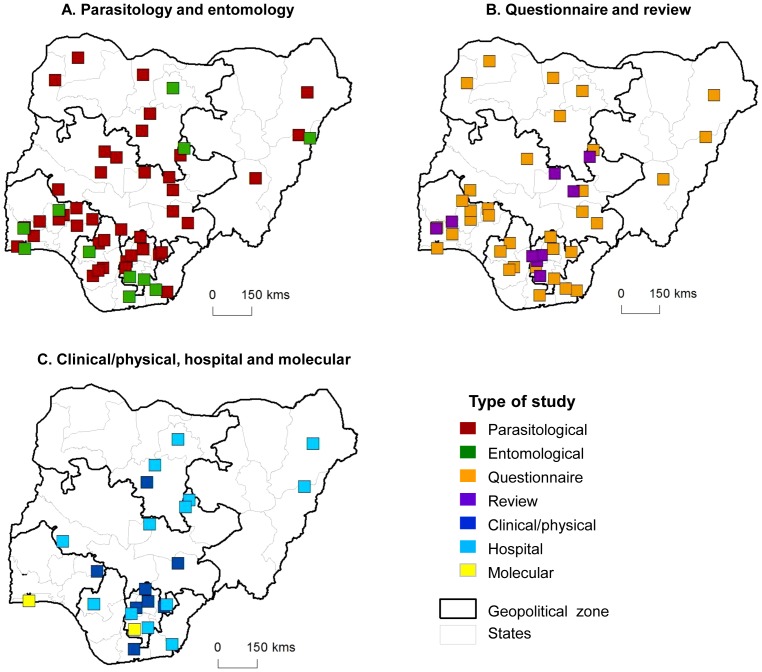
Institutional location and type of study. A. Parasitology and entomology. B. Social/anthropological and review. C. Clinical/physical, hospital and biotechnological.

Overall, the most common type of studies identified from the articles were parasitological (n = 214) and/or social/anthropological-based (n = 109), and the least common were biotechnological (n = 11) and reviews (n = 12). The type of studies carried out according to disease focus are shown in Table 2, and highlight that parasitological studies were the most common type of study for all diseases except for *L. loa* and one study on multiple diseases. The distribution of the study types varied across the country, with parasitological and social/anthropological-based studies widely distributed across all zones and most of the 31 states (Figure 3a). Entomological, clinical/physical examination and hospital based studies were carried out in all zones, however, the states varied; entomological (11 states), clinical/physical (15 states) and hospital based (13 states). Reviews were carried out in seven states of the North Central zone and in all three southern zones (South West, South East and South South), and biotechnological studies were only carried in two states of the South East (Imo State) and South West (Lagos State) zones (Figure 3b,c).

**Table 2:** Number of articles by study type for each main disease focus.

Disease focus	Biotechnological	Parasitological	Entomological	Social/anthropological	Clinical/Physical	Hospital	Reviews
LF	0	22	9	10	13	5	2
Onchocerciasis	3	21	13	18	19	2	3
Schistosomiasis	8	84	0	45	0	3	4
STH	0	79	0	28	2	12	1
Loa loa	0	2	0	5	0	0	0
Schistosomiasis, STH	0	6	0	3	0	1	4
LF, Loa loa, Onchocerciasis	0	0	0	0	0	0	1
**Total**	**11**	**214**	**22**	**109**	**34**	**23**	**12**

**Figure 4:**
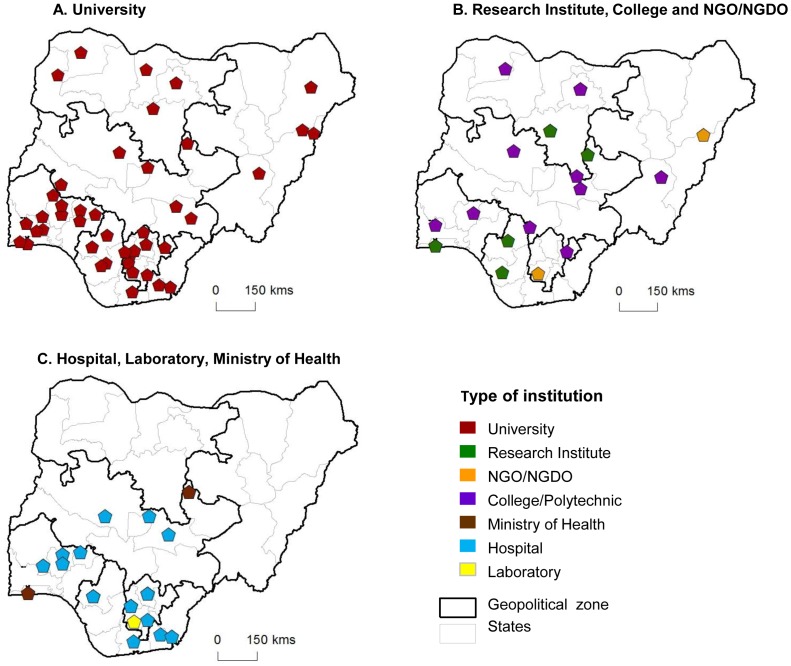
Institutional location and type of affiliation. A. University. B. Research Institute, College and NGO/NGDO. C. Hospital, Laboratory, Ministry of Health.

The articles published were from nine different types of institutions across the country (Table S1). Approximately ninety percent (89.6%) of the institutions were public owned while most of the hospitals (88.9%) were teaching hospitals. The most common type of institutional affiliation (of lead author) was the University (n = 198) accounting for two thirds (66.2%) of the total. Hospitals (n = 36) and Research Institutes (n = 20) represented 12% and 6.7% respectively, and authors from the Ministry of Health (n = 2) and NGO/NGDOs (n = 3) were found to lead least in research articles on these NTDs (Table 3). The trends were similar for the different diseases with the majority of authors being affiliated with a University however, there was some variation among the other institutional types, and notably, a higher proportion of STH articles were affiliated with hospitals (23.5%). The maps showing the distributions of the different institution types showed that the University affiliation was found in all zones and 29 of the states involved in NTD research (Figure 4a). Research Institute affiliations were found in North Central, North West, South South and South West zones across five states (Figure 4b), and hospital affiliations in the North Central and three southern zones (South West, South East and South South), across 13 states (Figure 4c).

**Table 3:** Number of articles by institutional type for each main disease focus.

Disease focus	University	Research Institute	College/Polytechnic	Diagnostic laboratory	Hospital	NGO/NGDO	Other*
LF	27	0	6	0	1	2	4
Onchocerciasis	39	8	1	0	5	0	4
Schistosomiasis	69	8	10	0	9	1	8
STH	53	3	0	1	19	0	4
Loa loa	5	0	0	0	1	0	0
Schistosomiasis, STH	4	1	0	0	1	0	0
LF, Loa loa, Onchocerciasis	1	0	0	0	0	0	0
**Total**	**198**	**20**	**18**	**1**	**36**	**3**	**20**

* Other includes internationally based lead author.

### International collaborators by disease focus and location

**Figure 5:**
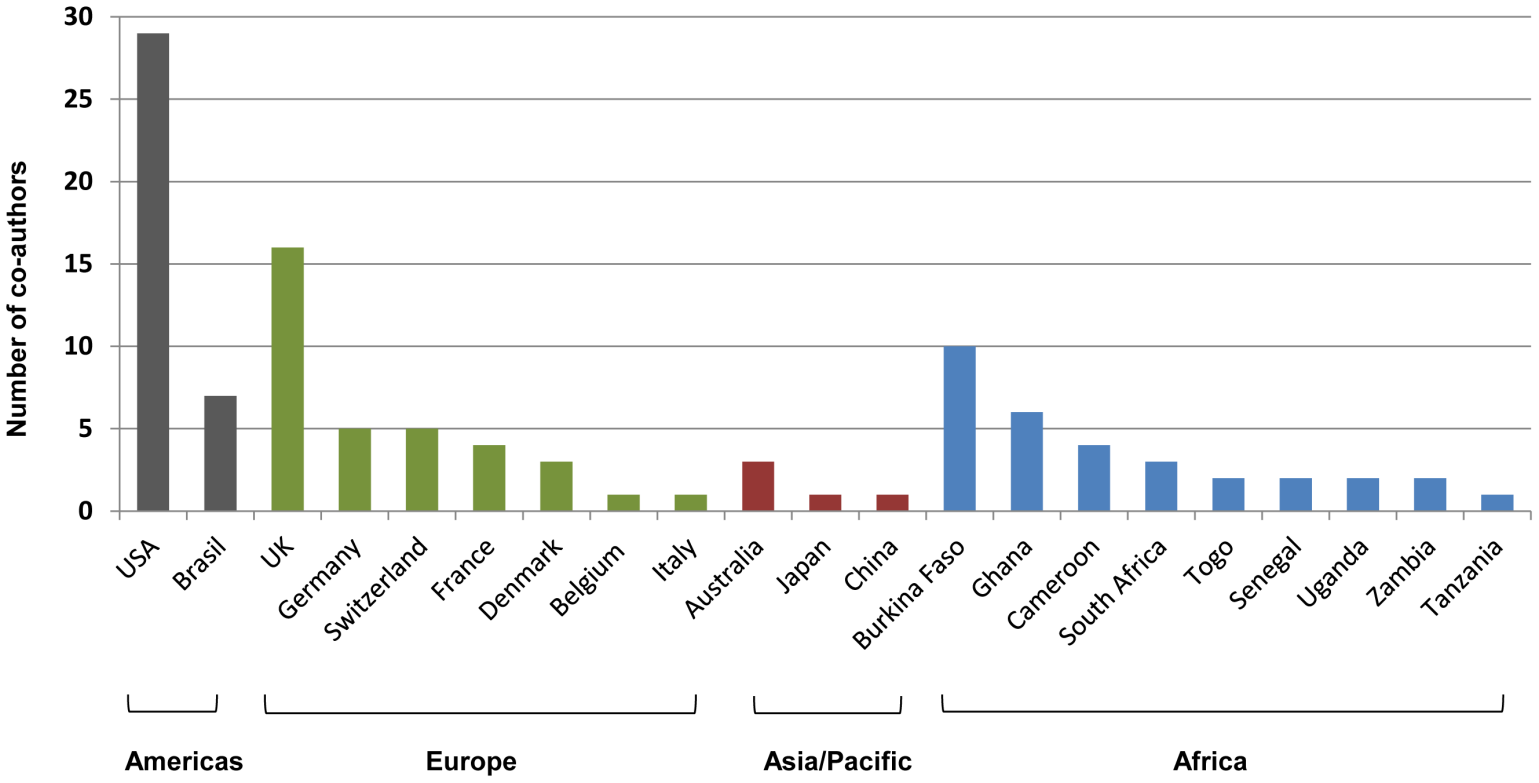
The number of international co-authors by country and global region.

A total of 45 (15.1%) of the 299 articles in the database were found to have international co-authors, with at least one Nigerian co-author included on the article (Table S1). In total, 192 international co-authors were tallied, with 29 international co-authors on LF articles (15.1%), 48 co-authors on onchocerciasis articles (25%), 85 co-authors on schistosomiasis articles (44.3%), 25 co-authors on STH articles (13%), none of *L.loa* and small number on articles published on multiple diseases. Overall, the international co-authors were from 22 countries from different regions of the world. The different countries were recorded 108 times on the 45 published articles (accounting for multiple international co-authors from same country). The most frequently cited country was USA (n = 29) followed by the UK (n = 13), Burkina Faso (n = 10) (reflecting a link with the African Programme for Onchocerciasis Control (APOC)), and Brasil (n = 7) with fewer co-authors reported from countries within Africa including Ghana (n = 6), Cameroon (n = 4), South Africa (n = 3), Togo (n = 2), Senegal (n = 2), Uganda (n = 2), Zambia (n = 2), Tanzania (n = 1), and elsewhere in Europe and the Asia-Pacific regions (Figure 5).

### Journal and impact factor by disease focus and collaborators

**Figure 6:**
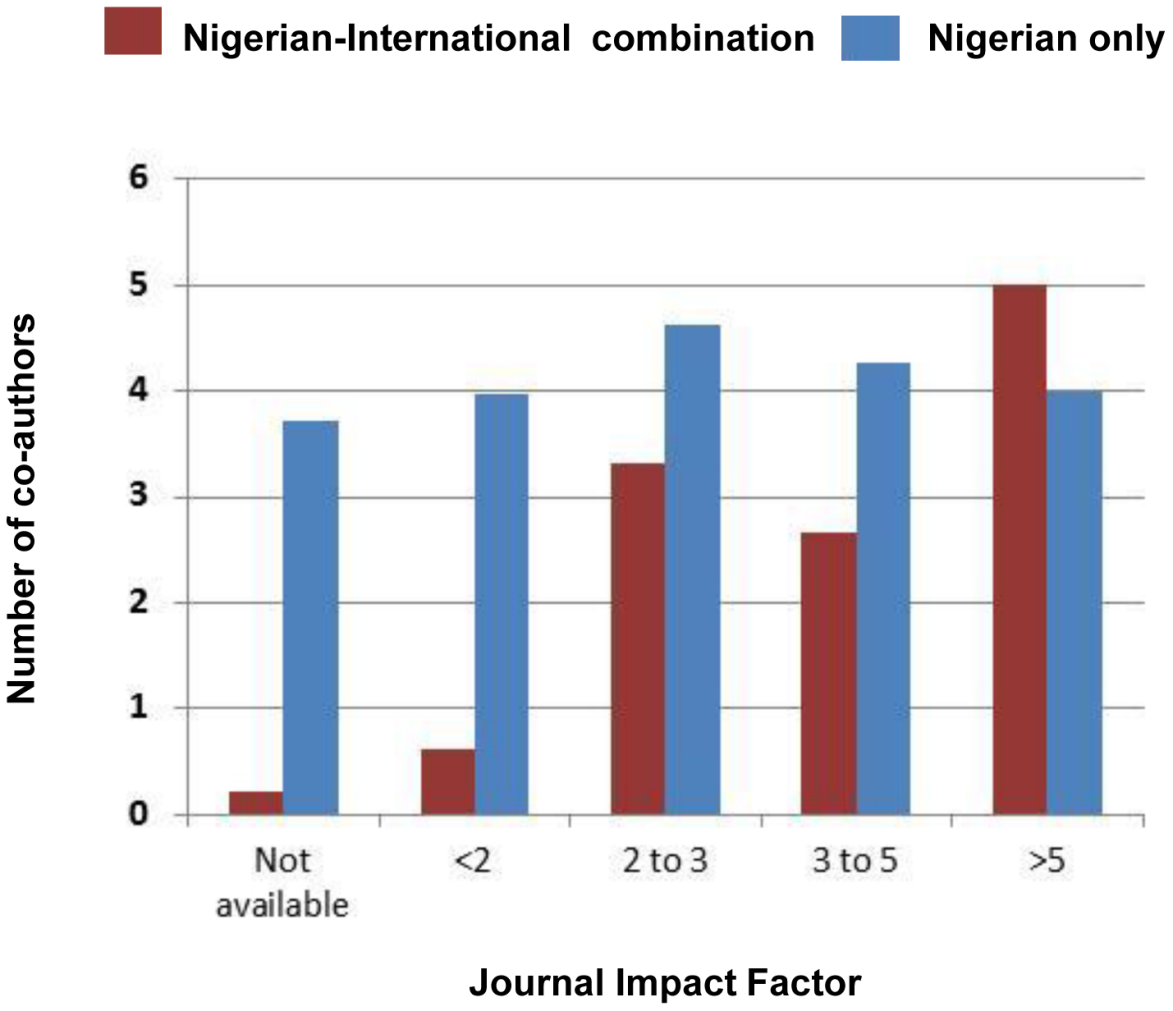
Average number of co-authors by journal impact factor.

The 299 articles were found to be published in 176 different journals, with 14 journals (8%) specifically with Nigerian research related titles. The Nigerian Journal of Parasitology had the highest number of publications (n = 18). The majority of articles (n = 209) were published in journals with no known impact factor (IF), while a total of 90 articles were in journals with IFs ranging from 0.25 to 5.85. The overall IF average was 1.99, and for LF articles was 2.43, onchocerciasis 1.89, schistosomiasis 2.16, STH 1.58 and *L. loa* 0.81. There were different trends between articles published by a combination of Nigerian-international co-authors and those by Nigerian authors alone, with a higher number of Nigerian-international co-authored articles published in journals with IFs over 2, than Nigerian-only authored articles which were published in journals with a wider range of IFs (Figure 6).

## Discussion

This critical analysis of research capacity in Nigeria highlights that there is a wide range of skilled researchers and institutions working on NTDs across many regions of the country. The relatively high number of publications indicates that there is significant potential to expand on and utilize this academic and technical human resource to collect, collate and map more epidemiological data, which are currently lacking [13]. The focus on publications over the past five years highlights the scientists who are most active at a time when the NTD momentum could best harness this local research capacity. This resource could also be used to help address specific NTD programmatic issues, and to highlight the barriers and successes to elimination across all diseases. It could also be used to enhance collaboration between the various institutions, donors, partners and international stakeholders, which is critical for Nigeria at the present time, given the emphasis on the scale up of implementation activities and integration of programmes.

There was a distinct difference between the number of publications by the geopolitical zones and states within them, with the highest research output coming from researchers in the southern zones. The classification or research output was based on location of researchers, hence, it may be possible that researchers may have carried out studies in other locations where they were not based. This north-south divide in NTD focus and capacity presented in this study, needs to be better understood to determine if it is related to the availability of resources, specific needs, opportunities, or even safety given the ongoing civil unrest in the northern zones [17]. Identifying these barriers and gaps is critical, and extending collaborative programmatic research activities to the north where there is significant co-endemicity of NTDs will be important, especially as it will likely be the region most difficult to eliminate them [13]. Addressing the elimination of NTDs in fragile conflict/post-conflict areas and countries is a significant challenge as they often have the highest disease burdens and least resources to cope [18]–[20].

The highest and most wide ranging research output was for schistosomiasis and STH. Notably however, most of the studies only focused on one disease, despite an extensive geographical and institutional overlap highlighted in our combined maps. A similar lack of integrated research was evident with LF and onchocerciasis, two diseases that often geographically overlap, have similar drug regimes and major programmatic barriers associated with the co-endemicity of *L. loa*. The lack of integrated disease research may be related to the fact that until recently, most NTD programmes and research groups had a more vertical disease-specific approach [21]–[24]. Now there is a significant national and international move to a horizontal or integrated programmatic approach with the development of NTD Master Plans, including mapping, implementing, and monitoring [25]. This programmatic paradigm shift will also affect future research, and has already started to be addressed in Nigeria [9]. For example, the FMoH recently launched a national guideline for the co-implementation of interventions to eliminate/control malaria and LF [26], which are both transmitted by the same *Anopheles* vectors in Nigeria [27].

The main type of research published was parasitological based (predominately microscopy), and those involving the use of questionnaire and focus group discussions. These types of studies are human resource intensive and do not require specialized or state-of the-art equipment, which may be the reason for their wide distribution. Researchers with these skills and experience could be an important human resource to the NTD Programmes, and help to collect and collate a range of epidemiological data across the country over time. Moreover the affiliated institutions could be used as a network of sites for training and/or conducting multi-centre and multi-disease studies [28] [29]. In contrast, there were a limited number of molecular based studies in the country, which suggests there is a need to increase access to specialized high-tech equipment and develop molecular and other laboratory skills through training programmes. This is increasingly important as technologies advance and specialized laboratory personnel could play a major role in the elimination process [30] [31].

Most articles were published by researchers affiliated with a University. In the Nigerian context, promotions for scientists and researchers, especially in the universities, are closely tied with publication output [32]–[35]. This in itself drives scientists to conduct and publish more research. This established academic infrastructure and human capacity, which is wide spread across the country, provides an opportunity for state level NTD programmes to link with local universities to conduct and publish operational research. This may increase the visibility of their work and help to inform other potential donors and international stakeholders of progress and challenges. It may also help to develop a collaborative network of programmatic-specific researchers within Nigeria, as well as externally between Nigerian and international scientists [6].

This study highlights a wide range of international collaborations and co-authors from Africa and other regions of the world, especially from the USA and UK. The impact of international research collaboration has not been extensively studied but could provide additional opportunities in terms of equipping laboratories, training scientists, providing mentorship, as well as helping to define an individual's career path, skills in grant writing, study design and publishing [2]. It was observed that articles with international co-authors, tended to be published in higher IF ranking journals. This trend has been reported elsewhere [36], and may translate to more international exposure and a wider readership, than those articles published in Nigerian journals alone. The importance of continuing and increasing international collaborations, including those within Africa (south south) and exposure to the work/research cannot be emphasized enough at a time when the country is scaling up efforts to eliminate multiple NTDs [37].

Although, it is possible that some papers by Nigerian authors may have been missed because they were not in the databases searched, this extensive geo-referenced database and series of maps on the research capacity within Nigeria, highlights the current potential that exists and can be built on for the control/elimination of NTDs in the country. This locally available capacity can be harnessed and strengthened by further investments (such as establishing up-to-date research facilities, conducting training activities, etc.) from the funding agencies/donors involved in NTD research and implementation in Nigeria. This database will promote an evidence-based approach to capacity strengthening and create the basis from which to build collaborative links within and outside the country as well as creating linkages across disciplines. This is important as Nigeria has recently been considered a priority country for early assistance to address the large burden of NTDs, and emphasizes the importance of providing clear, sound, and timely advice on technical aspects of programmatic activities [38].

Most importantly, this database provides an important model that can be applied to other low and middle income countries where NTDs are endemic. This study focussed only on the lead author information and the authors acknowledge that the FMoH have a lot of field capacity at state and local government area level which could also be utilised. However, the immediate country needs are to improve the knowledge and capacity of the State Ministry of Health staff responsible for the implementation of NTD programmes and through them at the Local Government Area (District) level. This will ensure that programmes supported by the State, bilateral donors and NGDOs are effectively implemented whilst research capacity is deployed to support the parallel implementation research needs, monitoring and evaluation and surveillance. The study demonstrates what is necessary in other countries and the urgency in building and developing capacity as stressed in the WHA Assembly resolution 66.12 of 2013 [4]. This is becoming critical in other highly populous countries of Africa, who are falling behind the curve as far as the WHO Road Maps targets are concerned, specifically in terms of the number of drugs that are required to be delivered to address what has become known as the “implementation deficit”.

## Supporting Information

Table S1 **Database of published papers on NTDs in Nigeria.**
(XLSX)

Checklist S1 **PRISMA checklist.**
(DOC)

Flow Diagram S1 **PRISMA flow diagram.**
(DOCX)
