# Supplementary material for: Neglected Tropical Diseases: A Systematic Evaluation of Research Capacity in Nigeria
Source: PLoS Negl Trop Dis. 2014 Aug 14;8(8):e3078. doi: 10.1371/journal.pntd.0003078 (PMC4133230; doi:10.1371/journal.pntd.0003078)
Supplement: Flow Diagram S1 — PRISMA flow diagram. (DOCX) [file pntd.0003078.s003.docx]

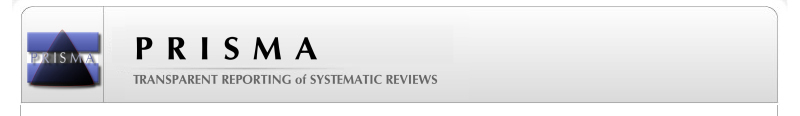
**PRISMA 2009 Flow Diag**

Studies included in quantitative synthesis (meta-analysis)
(n = 299 )

Studies included in qualitative synthesis
(n =299 )

Full-text articles excluded, with reasons
(n = 31 )

Full-text articles assessed for eligibility
(n = 321)

Records excluded
(n = 917 )

Records screened
(n = 321 )

Records after duplicates removed
(n = 1238 )

Additional records identified through other sources
(n = 254 )

## Identification

## Eligibility

## Included

## Screening

Records identified through database searching
(n = 1029 )
